# Arginine deprivation, growth inhibition and tumour cell death: 2. Enzymatic degradation of arginine in normal and malignant cell cultures

**DOI:** 10.1038/sj.bjc.6601096

**Published:** 2003-07-01

**Authors:** R Philip, E Campbell, D N Wheatley

## Abstract

**Correction to:** *British Journal of Cancer* (2003) **88**, 613–623; doi:10.1038/sj.bjc.66000681

Reference on p. 614 and in Table 2 (p. 615) to human recombinant arginine decarboxylase is incorrect, as elsewhere in the article. The enzyme was prepared by the overexpression of biosynthetic arginine decarboxylase (*spe*A), originating in *Escherichia coli* and purified from *E. coli* TOP 10 as a fusion protein (5xHis::speA) on a nickel-affinity column (Invitrogen).

We wish to apologise to Professor Stephen Boyle (Virginia Polytechnic Institute & State University, Blackburg, VA, USA), who kindly provided the enzyme for C*ancer Treatments International*, for this regrettable mistake.

DN Wheatley

